# Potential association of vacuum cleaning frequency with an altered gut microbiota in pregnant women and their 2-year-old children

**DOI:** 10.1186/s40168-015-0125-2

**Published:** 2015-12-21

**Authors:** Ekaterina Avershina, Anuradha Ravi, Ola Storrø, Torbjørn Øien, Roar Johnsen, Knut Rudi

**Affiliations:** Norwegian University of Life Sciences, Chemistry, Biotechnology and Food science department (IKBM), Campus Ås, Ås 1432 Ås, Norway; Department of Public Health and General Practice, Norwegian University of Science and Technology, 9491 Trondheim, Norway

## Abstract

**Background:**

Westernized lifestyle and hygienic behavior have contributed to dramatic changes in the human-associated microbiota. This particularly relates to indoor activities such as house cleaning. We therefore investigated the associations between washing and vacuum cleaning frequency and the gut microbiota composition in a large longitudinal cohort of mothers and their children. The gut microbiota composition was determined using 16S ribosomal RNA (rRNA) gene Illumina deep sequencing.

**Results:**

We found that high vacuum cleaning frequency about twice or more a week was associated with an altered gut microbiota composition both during pregnancy and for 2-year-old children, while there were no associations with house washing frequency. In total, six Operational Taxonomic Units (OTUs) showed significant False Discovery Rate (FDR) corrected associations with vacuum cleaning frequency for mothers (two positive and four negative) and five for 2-year-old children (four positive and one negative). For mothers and the 2-year-old children, OTUs among the dominant microbiota (average >5 %) showed correlation to vacuum cleaning frequency, with an increase in *Faecalibacterium prausnitzii* for mothers (*p* = 0.013, FDR corrected), and *Blautia* sp. for 2-year children (*p* = 0.012, FDR corrected).

**Conclusions:**

Bacteria showing significant associations are among the dominant gut microbiota, which may indicate indirect immunomodulation of the gut microbiota possibly through increased allergen (dust mites) exposure as a potential mechanism. However, further exploration is needed to unveil mechanistic details.

**Electronic supplementary material:**

The online version of this article (doi:10.1186/s40168-015-0125-2) contains supplementary material, which is available to authorized users.

## Background

Hygienic behavior and westernized lifestyle dramatically changes the way we are exposed to bacteria from the environment [1]. One of several factors related to the change into westernized lifestyle is increased indoor occupancy, with house washing and vacuum cleaning being the main hygienic activities. Cleaning activities may not only influence which bacteria we are exposed to but also how this exposure affects us. Vacuum cleaning has caused a particular attention with respect to increased exposure to allergens such as dust mites [2], and it has been shown that dust mite exposure has a potential impact on immunological status of the exposed subjects [3].

It has recently been established that the indoor environment microbiota is heavily associated with the families living there [4]. However, to our knowledge, no studies have yet addressed the association between indoor hygienic activities and the gut microbiota. This relation is important with respect to understanding the impact of the surrounding allergens on the gut microbiota [5].

The aim of our work was therefore to investigate the association between washing and vacuum cleaning and the gut microbiota for a large unselected cohort of mothers and their children. To study this, we reanalyzed a previously published longitudinal 16S ribosomal RNA (rRNA) gene mother-child gut microbiota dataset, where we have shown major age-related changes in the gut microbiota composition [6]. In the current study, we included the additional metadata information about house washing and vacuum cleaning. We have also included information about potential dietary confounding factors. We analyzed the associations with both alpha- and beta-diversity, in addition to the use of ANOVA-simultaneous component analysis (ASCA) [7] and Random Forest [8] to uncover potential complex metadata correlations in the longitudinal dataset.

We present results showing an association between vacuum cleaning and the gut microbiota both during pregnancy and in 2-year-old children.

## Methods

### Cohort description

IMPACT (Immunology and Microbiology in Prevention of Allergy among Children in Trondheim) study is a controlled non-randomized longitudinal study involving 720 groups of pregnant women and their children (up to 2 years of age). The majority of the children were vaginally delivered and at term (>90 %), with 97 % being breast-fed exclusively for the first 6 weeks of life [9]. Stool samples were collected during pregnancy, and at 10 days, 120 days, 1 year and 2 years, and stored in Cary-Blaire transport medium at −80 °C.

In the current study, samples from a subgroup of mother-child pairs (*n* = 356) with information about house washing and vacuum cleaning were included (Additional file 1: Table S1). We have overlapping information with microbiota data for (*n* = 82) pregnant women, (*n* = 63) 10-day-, (*n* = 85) 2-month-, (*n* = 75) 1-year- and (*n* = 68) 2-year-old children. We also included information about confounding dietary factors such as month for when rice, corn, wheat, bread, cooked vegetables, raw vegetables, fruits, commercial pre-made dinner, homemade dinner, fish, milk, or eggs were introduced for the first time (Additional file 1: Table S1). The information was obtained through questionnaires, as previously described [9].

### 16S rRNA gene dataset

We reanalyzed previously generated 16S rRNA gene data [6]. These data were generated by PCR amplification using primers targeting universally conserved regions of the 16S rRNA gene flanking the variable regions V3 and V4 [10], with DNA isolated from mechanically lysed cells as template. Sequencing was done using the Illumina MiSeq platform with the V3 chemistry. The resulting data were analyzed using QIIME [11]. Sequences were quality filtered (*split_libraries.py*; sequence length between 200 bp and 1000 bp; minimum average quality score 25; not more than 6 ambiguous bases; no primer mismatch allowed) and then clustered at 99 % homology level using closed-reference *uclust* search against Greengenes database [12] (*pick_closed_reference_otus.py*). Finally, 4000 sequences per sample were randomly picked from the full dataset to unify amount of information for each sample. The resulting Operational Taxonomic Unit (OTU) table contained 6920 OTUs for a total of 373 samples.

### Data analyses

We used Simpson’s index to investigate alpha-diversity and Variance Weighted Distance Between Cluster Centers (Ward’s) based on an Euclidean distance matrix to determine beta-diversity. To uncover potential complex associations between metadata and the gut microbiota ASCA [7], Random Forest [8] analyses with the OTU table as response and cleaning frequency (washing and vacuum cleaning frequency binarized with respect to the median) as predictor were used. The rationale of using median binarization of the data is to increase the power of the analyses and to determine whether the overall associations of the microbiota with the predictor variables are statistically significant. To investigate the direct OTU associations with the predictor variables, we used Kruskal-Wallis non-parametric one-way analysis of variance, in addition to Spearman non-parametric correlations for dose response analyses. We used False Discovery Rate (FDR) to correct for multiple testing [13].

Basic statistical analyses were done using Minitab 16 (Minitab Inc, USA), while multivariate analyses were done using the PLS Toolbox (Eigenvector Inc, USA) plugin in the MATLAB® R2014a (Mathworks, USA) environment. For phylogenetic visualization, we used Itol (itol.embl.de).

## Results

### Cleaning

In the IMPACT study, there were 358 mother-child pairs with complete information about monthly cleaning and vacuum cleaning frequency. The cleaning frequencies were generally stable throughout the period investigated from late pregnancy until the child was 2 years (Additional file 1: Table S1), with an average frequency of 2.9 washings and 6.6 vacuum cleanings per month. We found a slight positive correlation between washing and vacuum cleaning frequencies (*R*^2^ = 0.13, *p* < 0.001, Pearson), while there were very minor or no correlations between cleaning and introduction of rice, corn, wheat, bread, cooked vegetables, raw vegetables, fruits, commercial pre-made dinner, homemade dinner, fish, milk, or eggs into infants’ diet (*R*^2^ < 0.01, *p* > 0.05, Pearson).

### Association between cleaning frequency and the gut microbiota

**Fig. 1:**
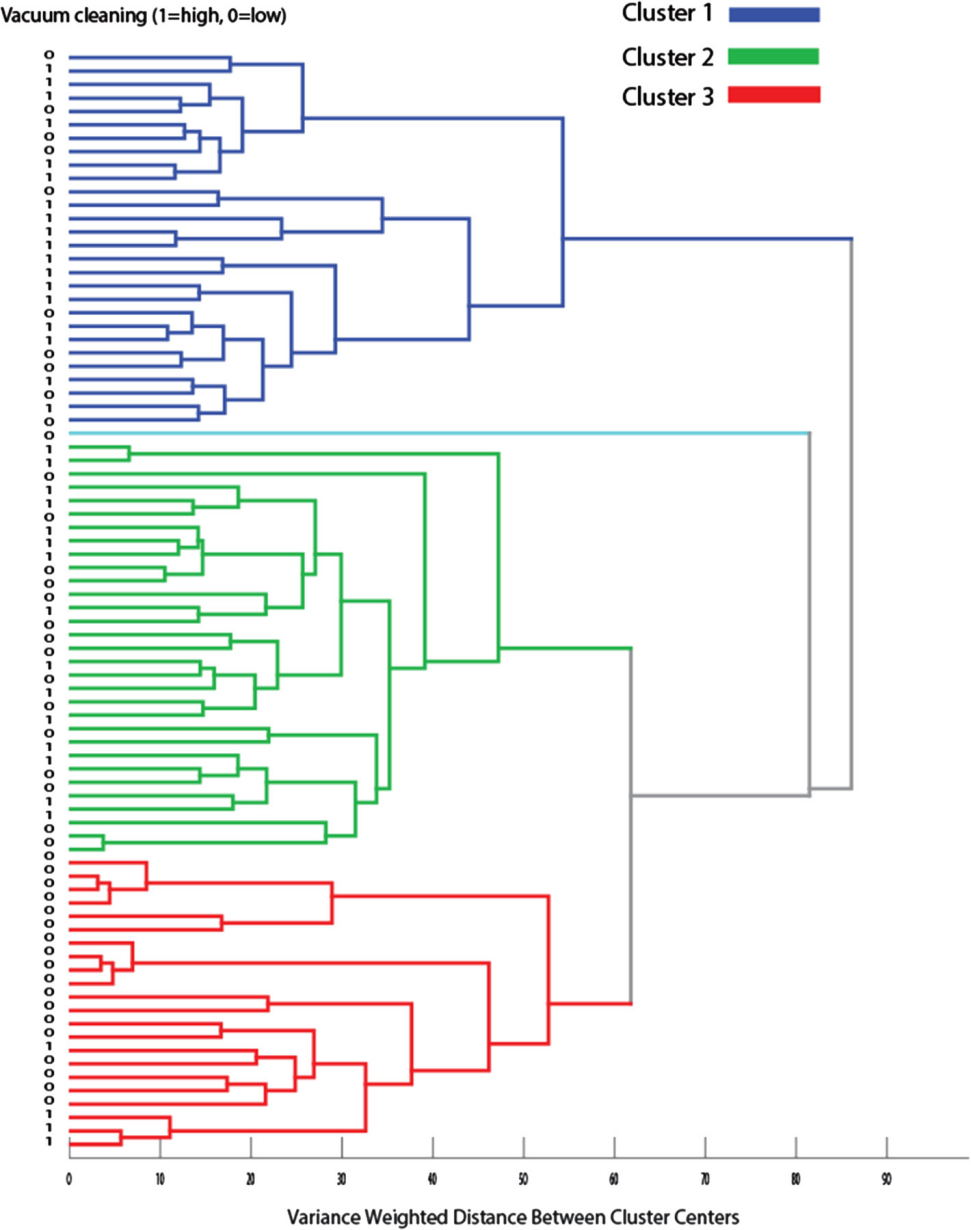
Association between beta-diversity and vacuum cleaning for pregnant mothers. The *color code* represents the three main clusters detected by Ward’s analyses based on Euclidean distances for the OTU abundance data

We found no significant associations for alpha-diversity for any of the age categories, but for beta-diversity, we found a significant association. Based on the microbiota composition, the mothers clustered into three distinct clusters (Cluster 1 to 3; Fig. 1), where Cluster 1 showed association with high vacuum cleaning frequency and Cluster 3 with low (*p* < 0.0005, likelihood ratio chi-square test).

**Fig. 2:**
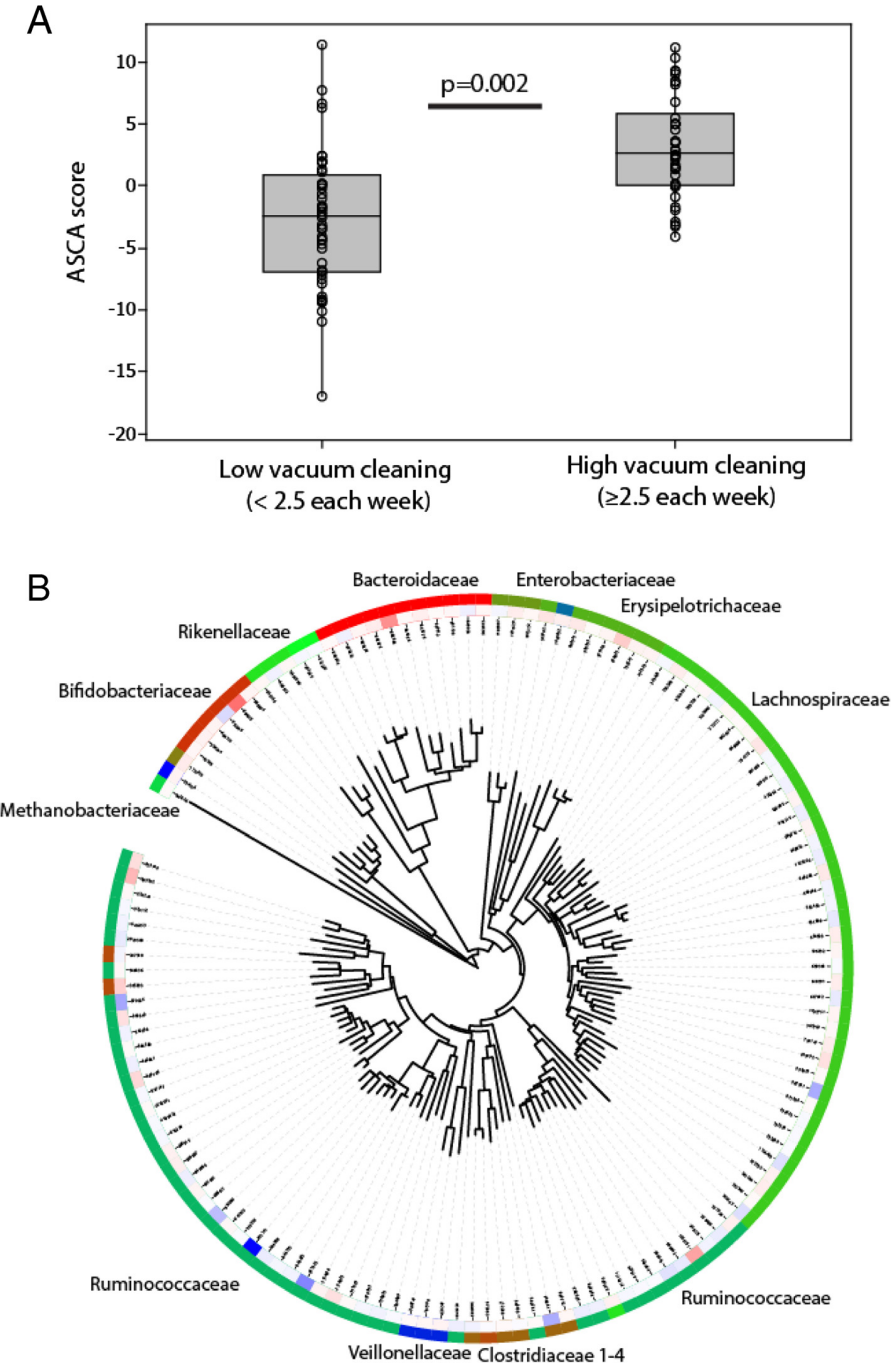
Association between the pregnant mother’s microbiota and vacuum cleaning frequency determined by ASCA analyses. **a** Overall association of microbiota with vacuum cleaning. **b** Phylogenetic association of the OTUs contributing to explaining the differences in the microbiota. The *inner circle* indicates the importance of the OTUs, where *blue* indicates positive association and *red* negative (ASCA loads spanning ±0.6)

**Fig. 3:**
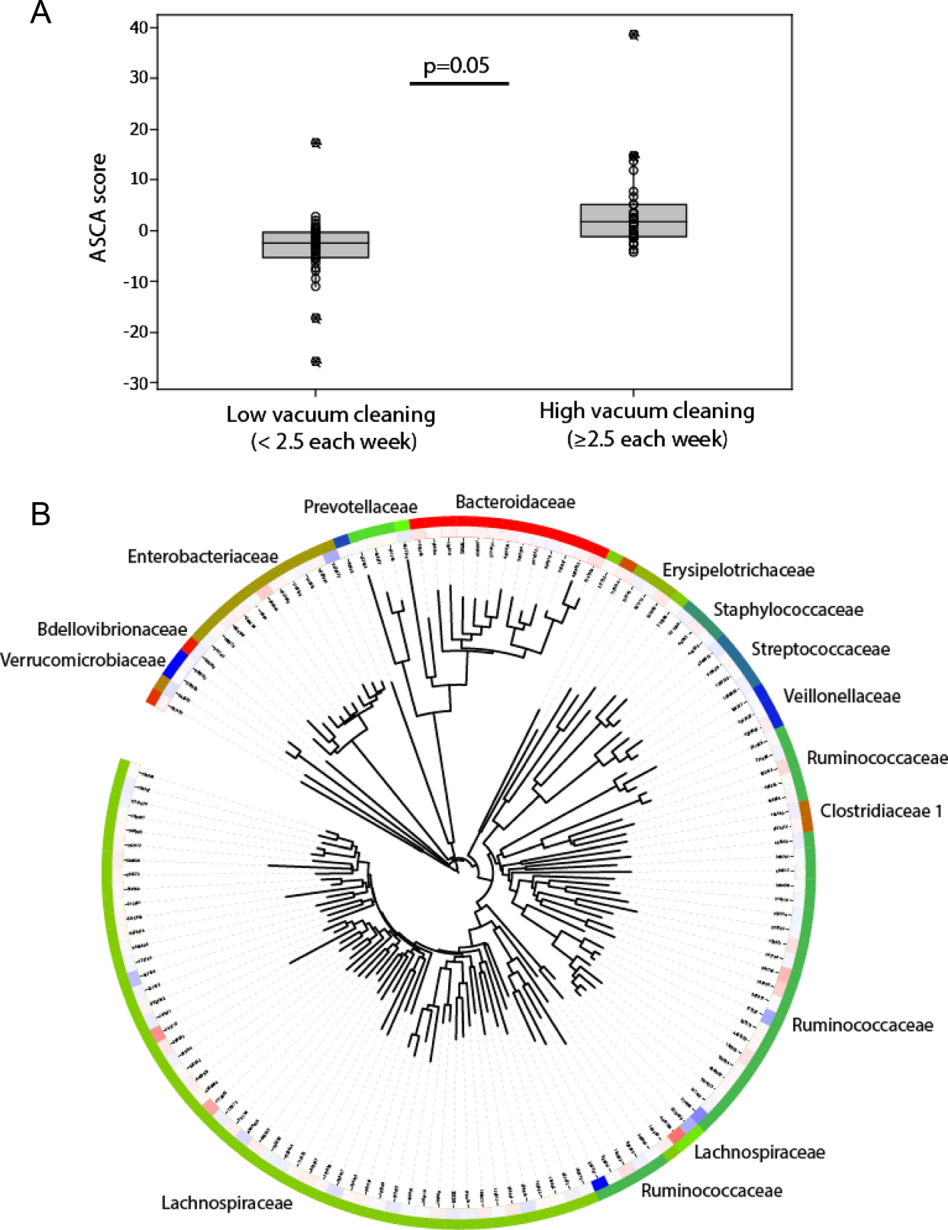
Association between the 2-year microbiota and vacuum cleaning determined by ASCA analyses. **a** Overall association of microbiota with vacuum cleaning. **b** Phylogenetic association of the OTUs contributing to explaining the differences in the microbiota. The *inner circle* indicates the importance of the OTUs, where *blue* indicates positive association and *red* negative (ASCA loads spanning ±0.6)

For the compositional association between the microbiota and cleaning frequencies by ASCA, we found no significant associations for washing, while we detected significant associations for vacuum cleaning at pregnancy and 2-year children (Figs. 2a and 3a, respectively). However, only a few OTUs were important for these associations (Figs. 2b and 3b, respectively). Random Forest revealed a significant discrimination between high- and low vacuum cleaning frequency only for mothers (*p* = 0.007, Kruskal-Wallis test), while for the 2-year old, this discrimination was at the border of significance (*p* = 0.058; Kruskal-Wallis test). As for the ASCA, only a few OTUs were influential in the models (Additional file 2: Figure S1).

At pregnancy, OTU851141 related to *Faecalibacterium prausnitzii* showed the strongest positive influence on the ASCA model for vacuum cleaning. This OTU also showed a significant direct association with vacuuming (median 6.3 % (high) vs 1.8 % (low), *p* = 0.006 Kruskal-Wallis test). OTU567381 related to *Roseburia faecis* was identified as the most influential for Random Forest. This OTU also showed significant direct association with vacuum cleaning frequency (median 1.2 % (high) vs 0.33 % (low), *p* = 0.003 Kruskal-Wallis test). As for the negative associations during pregnancy, only OTU584375 related to *Bifidobacterium adolescentis* was detected as influential by ASCA, with a median of 2.7 % for low vacuum cleaning and 1.3 % for high vacuum cleaning frequency (*p* = 0.034, Kruskal-Wallis test). For the 2-year-olds, OTU1104433 classified as *Blautia* sp. was the most influential as determined by ASCA (median 4.9 %, vs 2.8 % for high and low vacuum cleaning frequency, respectively; *p* = 0.015 Kruskal-Wallis test), while OTU844941 related to *Oscillospira* sp. was identified by Random Forest (median 0.23 % vs 0.05 % for high and low vacuum cleaning frequency, respectively; *p* = 0.001, Kruskal-Wallis test).

To determine potential quantitative associations, we investigated the OTUs directly correlated with vacuum cleaning after FDR correction for mothers and 2-year-old children using Spearman non-parametric correlation. We identified the same positively associated OTUs, as identified by ASCA and Random Forest, while the negatively associated OTU identified by ASCA did not show significance. In addition to the ASCA and Random Forest identified OTUs, a set of low abundant OTUs were also identified for the Spearman correlations (Table 1).

**Table 1:** Direct correlation of OTUs for dust cleaning mother and 2-year children

OTU#	Origin	Average abundance (%)	Spearman regression coeff	FDR corrected *p* value	Family	Genus	Species
851141	Mother	5.61	0.28	0.013	*Ruminococcaceae*	*Faecalibacterium*	*Prausnitzii*
567381	Mother	1.12	0.29	0.008	*Lachnospiraceae*	*Roseburia*	*Faecis*
672795	Mother	1.06	−0.25	0.025	*Lachnospiraceae*	*Blautia*	
365893	Mother	0.44	−0.28	0.011	*Ruminococcaceae*		
805192	Mother	0.43	−0.23	0.046	*Lachnospiraceae*	*Roseburia*	
369166	Mother	0.40	−0.28	0.011	*Ruminococcaceae*		
1104433	Child 2 years	6.31	0.32	0.012	*Lachnospiraceae*	*Blautia*	
364609	Child 2 years	0.38	0.29	0.027	*Ruminococcaceae*		
844941	Child 2 years	0.21	0.37	0.002	*Ruminococcaceae*	*Oscillospira*	
369970	Child 2 years	0.20	0.31	0.012	*Lachnospiraceae*	*Ruminococcus*	
839512	Child 2 years	0.15	−0.33	0.010	*Bacteroidaceae*	*Bacteroides*	*Eggerthii*

## Discussion

Mothers clustered into three distinct clusters with respect to beta-diversity with one of these clusters associated with high vacuum cleaning frequency, while another was associated with low frequency. It could therefore be that household environment and hygienic behavior can be a potential contributing factors to the overall clustering pattern observed for the adult gut microbiota [14].

Vacuum cleaning can lead to increased allergen exposure through dust mites [2], and it has recently been shown that dust mite associated Toll-like receptor 4 (TLR4) signaling has an important role for inflammation in the airways in the body [3]. Therefore, it is plausible that immunological signaling in the airway mucosa could affect the gut mucosa through a common signaling system [15] and consequently the associated gut microbiota. In concurrence, we identified correlations for vacuum cleaning and not for house washing which supports a potential importance of airway exposure for gut microbiota modulation.

For mothers, we found the largest increase of *F. prausnitzii* at high vacuum cleaning frequency. This bacterium is anti-inflammatory [16], harvesting energy through extracellular electron transport [17]. Therefore, inflammation-induced reactive oxygen could be an energy source for *F. prausnitzii* leading to an expansion of the population, while its anti-inflammatory properties could potentially counteract an inflammatory response. We also identified a relatively large decrease in *B. adolescentis,* which is in line with its previously observed negative association with TLR4 induction [18].

There was no overlap in the OTUs associated with vacuum cleaning for mothers and the 2-year children. A potential explanation could be that the associations for the 2-year children are due to vacuum cleaning-associated immunological/microbiota differences in mothers during pregnancy [19]. *Blautia* sp. showed the most pronounced positive association with vacuum cleaning for the 2-year children, while a negative association was detected for the mothers. This bacterial group shows a high degree of host specificity [20, 21] and is one of the main acetogens in the gut, harvesting energy by assimilation of carbon dioxide and hydrogen [22]. Still, however, our knowledge is too limited to mechanistically link *Blautia* to vacuum cleaning.

The association between microbiota and vacuum cleaning could also be confounded by unknown factors. A factor not included here is the use of High-Efficiency Particulate Arrestance (HEPA) filters in vacuum cleaners and differences between vacuum cleaners with respect to particle release. Whether or not, the level of particle release is confounded with vacuum cleaning frequency remains unknown. For the dietary factors measured, there were no or only minor associations with vacuum cleaning. Therefore, these are unlikely confounders with respect to the observed association between the gut microbiota and vacuum cleaning. However, there may of course be other confounding factors not covered here.

ASCA and Random Forest identified different parts of the microbiota, with ASCA identifying the most dominant, while Random Forest identified the most discriminative. Since ASCA is a generalization of ANOVA from univariate to multivariate data [7], it is expected that this approach will identify dominant OTUs. Random Forest, on the other hand, is a machine-learning approach aimed at identifying any associations between predictor and response variables [23]. This is probably the reason why Random Forest was more sensitive to low abundant OTUs. Therefore, ASCA and Random Forest seem complementary in relating the microbiota to environmental factors through both the dominant and non-dominant part of the microbiota.

## Conclusions

In conclusion, our data point toward the possibility of gut microbiota modulation through airway allergen exposure. Thus, this could potentially add an additional facet to the complexity of the human gut microbiota interactions with the host.

## Additional files

Additional file 1: Table S1. Metadata for cleaning and diet. The table shows detailed metadata for cleaning and diet. The data are self-reported. (DOC 619 kb) Additional file 2: Figure S1. Out-of-bag evaluation of OTU importance by Random Forest for (A) mothers and (B) 2-year children. The models were based leave sizes of 10 for ensembles of 100 trees. The leave size was determined by the criterion of minimum mean squared error. (DOC 51 kb)
