# Supplementary material for: Potential association of vacuum cleaning frequency with an altered gut microbiota in pregnant women and their 2-year-old children
Source: Microbiome. 2015 Dec 21;3:65. doi: 10.1186/s40168-015-0125-2 (PMC4685603; doi:10.1186/s40168-015-0125-2)
Supplement: Additional file 1: Table S1. — Metadata for cleaning and diet. The table shows detailed metadata for cleaning and diet. The data are self-reported. (DOC 619 kb) [file 40168_2015_125_MOESM1_ESM.doc]

**Additional file 1. Suppl. Table 1. Metadata for cleaning and diet**

| **mother`/child pair** | **house washed times per month (pr)** | **house vacuum-cleaned times per month (pr)** | **house washed times per month (6w)** | **house vacuum-cleaned times per month (6w)** | **house washed per month(1y)** | **house vacuum-cleaned per month(1y)** | **rice first time, mo** | **corn first time, mo** | **wheat first time, mo** | **bread first time, mo** | **cooked veg first time, mo** | **raw veg first time, mo** | **fruits first time, mo** | **commercial pre-made dinner first-time, mo** | **home-made dinner first time, mo** | **fish first time, mo** | **milk first time, mo** | **eggs first time, mo** |
| --- | --- | --- | --- | --- | --- | --- | --- | --- | --- | --- | --- | --- | --- | --- | --- | --- | --- | --- |
| 1278 | 3 | 0 |  |  |  |  |  |  |  |  |  |  |  |  |  |  |  |  |
| 1375 | 4 | 0 |  |  |  |  |  |  |  |  |  |  |  |  |  |  |  |  |
| 1391 | 5 | 0 | 5 | 0 | 5 | 0 | 5 | 5 | 6 | 7 | 7 | 8 | 8 | 7 | 7 | 12 | 11 |  |
| 2449 | 4 | 0 |  |  |  |  |  |  |  |  |  |  |  |  |  |  |  |  |
| 1146 | 4 | 1 | 4 | 0 |  |  |  |  |  |  |  |  |  |  |  |  |  |  |
| 1356 | 4 | 0 | 4 | 0 | 2 | 2 | 8 |  | 6 | 5 | 4 | 12 | 4 |  | 4 | 6 | 6 | 5 |
| 1645 | 1 | 2 | 3 | 0 | 2 | 0 | 5 |  |  | 8 | 4 | 11 | 4 |  | 7 |  | 11 |  |
| 2556 | 4 | 1 | 2 | 1 | 4 | 0 | 4 |  | 6 | 6 | 6 | 18 | 6 | 6 | 6 | 12 | 12 | 12 |
| 211 | 2 | 2 | 2 | 1 | 2 | 0 |  | 6 | 7 | 8 | 8 |  | 6 | 8 | 8 |  | 12 | 12 |
| 1454 | 4 | 1 | 4 | 0 | 4 | 2 | 5 | 5 | 6 | 7 | 9 | 10 | 5 | 6 | 7 | 11 | 11 |  |
| 1831 | 2 | 1 |  |  |  |  |  |  |  |  |  |  |  |  |  |  |  |  |
| 1864 | 2 | 1 |  |  |  |  |  |  |  |  |  |  |  |  |  |  |  |  |
| 1898 | 3 | 1 | 2 | 0 | 1 | 2 | 6 | 6 | 7 | 9 | 9 |  | 9 |  | 10 | 11 | 12 |  |
| 2578 | 2 | 1 |  |  |  |  |  |  |  |  |  |  |  |  |  |  |  |  |
| 1720 | 4 | 1 | 4 | 2 | 4 | 1 |  |  | 6 | 6 | 7 | 10 | 7 | 8 | 8 | 12 | 12 |  |
| 2549 | 2 | 1 | 2 | 2 | 2 | 1 | 12 | 6 |  | 5 | 5 |  | 5 | 5 | 5 | 5 | 11 |  |
| 1930 | 3 | 2 | 4 | 1 |  |  |  |  |  |  |  |  |  |  |  |  |  |  |
| 2430 | 1 | 2 | 1 | 1 |  |  |  |  |  |  |  |  |  |  |  |  |  |  |
| 115 | 3 | 2 | 3 | 2 | 2 | 2 |  |  | 5 | 8 |  |  |  | 4 |  |  |  |  |
| 116 | 4 | 2 |  |  | 2 | 2 | 5 | 5 | 7 | 10 | 8 |  | 7 | 7 | 10 |  | 11 |  |
| 1134 | 2 | 2 |  |  |  |  |  |  |  |  |  |  |  |  |  |  |  |  |
| 1251 | 4 | 2 |  |  |  |  |  |  |  |  |  |  |  |  |  |  |  |  |
| 1270 | 2 | 2 |  |  |  |  |  |  |  |  |  |  |  |  |  |  |  |  |
| 1504 | 2 | 2 | 2 | 2 | 2 | 2 | 5 | 5 | 6 | 9 | 6 | 7 | 6 | 6 | 6 | 8 | 7 | 7 |
| 1636 | 2 | 2 | 2 | 2 | 2 | 2 | 4 | 4 | 4 | 9 | 5 | 10 | 10 | 5 | 5 | 11 | 10 |  |
| 1640 | 4 | 2 |  |  |  |  |  |  |  |  |  |  |  |  |  |  |  |  |
| 1651 | 2 | 2 |  |  |  |  |  |  |  |  |  |  |  |  |  |  |  |  |
| 1660 | 1 | 2 | 1 | 2 | 1 | 2 | 6 | 6 | 6 | 7 | 6 |  | 5 | 7 |  |  | 12 |  |
| 1807 | 1 | 2 |  |  |  |  |  |  |  |  |  |  |  |  |  |  |  |  |
| 1933 | 3 | 1 | 2 | 1 | 4 | 4 |  | 6 | 6 | 10 | 10 |  | 7 | 7 | 10 | 10 | 11 |  |
| 1940 | 2 | 2 |  |  |  |  |  |  |  |  |  |  |  |  |  |  |  |  |
| 1993 | 4 | 2 |  |  |  |  |  |  |  |  |  |  |  |  |  |  |  |  |
| 2063 | 2 | 2 | 2 | 2 |  |  |  |  |  |  |  |  |  |  |  |  |  |  |
| 2078 | 2 | 2 | 2 | 2 | 2 | 2 | 4 | 4 | 8 | 6 | 10 | 10 | 6 | 8 | 12 | 11 | 12 |  |
| 2553 | 2 | 2 | 1 | 2 |  |  |  |  |  |  |  |  |  |  |  |  |  |  |
| 2573 | 2 | 1 | 2 | 4 |  |  |  |  |  |  |  |  |  |  |  |  |  |  |
| 1238 | 1 | 2 | 2 | 2 | 3 | 4 | 5 |  | 5 | 8 | 8 |  | 5 | 5 | 10 | 12 | 10 |  |
| 1294 | 2 | 3 | 2 | 4 | 2 | 1 | 11 | 6 | 7 | 7 | 6 |  | 6 | 6 | 6 | 11 | 11 |  |
| 2151 | 2 | 2 | 1 | 2 | 3 | 4 |  | 5 | 5 | 7 | 7 |  | 6 | 7 |  | 9 | 12 |  |
| 20069 | 3 | 3 | 2 | 2 | 2 | 3 | 4 | 4 |  | 6 | 4 | 10 | 6 | 6 | 6 | 12 | 10 | 10 |
| 336 | 4 | 4 | 3 | 2 |  |  |  |  |  |  |  |  |  |  |  |  |  |  |
| 1244 | 2 | 3 |  |  |  |  |  |  |  |  |  |  |  |  |  |  |  |  |
| 1269 | 4 | 4 |  |  |  | 2 | 4 | 4 | 5 | 6 | 6 | 7 | 6 | 6 | 6 | 6 | 12 |  |
| 1299 | 1 | 3 |  |  |  |  |  |  |  |  |  |  |  |  |  |  |  |  |
| 1302 | 2 | 3 | 3 | 4 | 1 | 2 |  | 5 | 5 | 9 | 5 | 8 | 6 | 6 | 5 | 7 | 9 | 12 |
| 1359 | 3 | 3 |  |  |  |  |  |  |  |  |  |  |  |  |  |  |  |  |
| 1369 | 3 | 2 | 2 | 4 |  |  |  |  |  |  |  |  |  |  |  |  |  |  |
| 1381 | 2 | 3 | 3 | 3 |  |  |  |  |  |  |  |  |  |  |  |  |  |  |
| 1520 | 2 | 3 |  |  |  |  |  |  |  |  |  |  |  |  |  |  |  |  |
| 1659 | 1 | 3 |  |  |  |  |  |  |  |  |  |  |  |  |  |  |  |  |
| 1926 | 3 | 3 | 3 | 3 |  |  |  |  |  |  |  |  |  |  |  |  |  |  |
| 1954 | 2 | 2 |  |  | 2 | 4 | 4 | 4 | 8 | 7 | 1 | 1 | 5 | 6 | 1 | 10 |  |  |
| 1970 | 2 | 4 |  |  | 2 | 2 | 5 | 6 | 6 | 6 | 5 | 6 | 6 | 6 | 6 | 10 | 11 |  |
| 1992 | 2 | 3 |  |  |  |  |  |  |  |  |  |  |  |  |  |  |  |  |
| 2040 | 4 | 6 | 5 | 2 | 9 | 1 |  | 4 | 6 | 9 | 7 | 16 | 7 | 6 | 6 | 12 | 12 | 12 |
| 2058 | 2 | 3 | 2 | 2 | 2 | 4 | 5 |  | 8 | 9 | 9 | 10 | 9 | 6 | 6 | 12 | 10 |  |
| 2376 | 3 | 3 | 2 | 4 | 2 | 2 | 4 |  | 4 | 6 | 5 | 9 | 5 | 4 | 4 | 5 | 8 |  |
| 2543 | 2 | 3 |  |  |  |  |  |  |  |  |  |  |  |  |  |  |  |  |
| 2551 | 3 | 4 | 2 | 3 | 3 | 2 | 5 | 5 | 8 | 8 | 5 | 10 | 6 | 5 | 6 | 10 | 11 | 12 |
| 1265 | 1 | 4 | 1 | 4 | 1 | 2 | 10 | 6 | 6 | 10 | 10 | 19 | 10 | 4 | 8 | 10 | 10 | 10 |
| 1522 | 3 | 3 | 2 | 4 | 1 | 3 | 5 | 5 | 6 | 7 | 8 |  | 5 | 6 | 6 | 10 |  |  |
| 1913 | 1 | 2 | 2 | 4 | 2 | 4 |  | 4 | 6 | 6 |  |  | 4 | 4 | 7 | 10 | 8 | 6 |
| 1960 | 4 | 4 | 3 | 3 | 2 | 3 | 8 | 8 | 8 | 6 | 10 | 10 | 6 | 6 | 10 | 11 | 11 |  |
| 1997 | 2 | 4 | 2 | 2 | 2 | 4 | 5 | 6 | 6 |  | 6 |  | 6 | 6 | 6 |  |  |  |
| 2124 | 4 | 4 | 4 | 2 | 4 | 4 | 12 | 6 | 6 | 8 | 6 | 14 | 6 | 6 | 6 | 6 | 12 | 16 |
| 2425 | 3 | 3 | 3 | 3 | 3 | 4 | 6 | 6 | 7 | 10 | 7 | 9 | 6 | 10 | 6 | 8 | 12 | 12 |
| 303 | 3 | 3 |  |  | 3 | 4 | 3 | 3 | 64 | 6 | 6 | 10 | 6 | 8 | 8 | 7 | 11 | 12 |
| 1220 | 4 | 3 |  |  | 4 | 4 |  |  | 5 | 7 | 6 |  | 6 |  | 6 | 7 |  | 11 |
| 1317 | 4 | 6 |  |  | 4 | 1 |  |  | 5 | 8 | 8 | 11 | 9 | 7 | 12 | 12 | 12 | 12 |
| 1472 | 3 | 3 | 3 | 4 | 3 |  | 4 | 4 | 4 | 8 | 6 |  | 6 | 4 | 6 | 7 | 10 |  |
| 1483 | 3 | 3 | 4 | 4 |  |  |  |  |  |  |  |  |  |  |  |  |  |  |
| 1875 | 1 | 3 | 1 | 4 |  |  |  |  |  |  |  |  |  |  |  |  |  |  |
| 1617 | 2 | 4 | 2 | 5 | 1 | 2 | 6 | 6 | 7 | 8 | 6 | 8 | 6 | 8 | 6 | 10 | 11 |  |
| 1966 | 1 | 2 | 2 | 3 | 2 | 6 |  |  |  |  |  |  |  |  |  |  |  |  |
| 2048 | 2 | 4 | 2 | 4 | 1 | 3 | 4 | 4 | 6 | 8 | 4 | 11 | 6 | 4 | 9 | 10 | 12 | 12 |
| 2223 | 4 | 3 | 3 | 4 | 3 | 4 |  | 5 | 6 | 9 | 5 |  | 5 | 7 | 5 | 6 | 11 |  |
| 38 | 4 | 4 |  |  |  |  |  |  |  |  |  |  |  |  |  |  |  |  |
| 112 | 1 | 3 | 2 | 5 | 2 | 4 |  |  | 3 | 6 | 11 |  | 7 | 5 | 10 | 10 | 6 | 10 |
| 197 | 3 | 4 | 3 | 4 | 4 | 4 | 5 | 6 | 6 | 10 | 6 | 12 | 12 | 6 | 6 | 6 | 12 | 16 |
| 212 | 2 | 4 | 1 | 5 | 3 | 3 | 9 | 4 | 4 | 6 | 6 | 8 | 7 | 6 | 6 | 5 | 8 |  |
| 309 | 1 | 4 | 2 | 4 |  |  |  |  |  |  |  |  |  |  |  |  |  |  |
| 924 | 2 | 4 |  |  | 2 | 4 | 7 | 7 | 9 | 9 | 9 |  | 6 | 9 | 9 |  |  |  |
| 1136 | 5 | 4 |  |  |  |  |  |  |  |  |  |  |  |  |  |  |  |  |
| 1145 | 4 | 8 | 16 | 0 |  |  |  |  |  |  |  |  |  |  |  |  |  |  |
| 1285 | 4 | 4 | 2 | 4 | 4 | 4 | 5 | 5 | 7 |  | 6 |  | 5 | 6 |  |  |  |  |
| 1308 | 4 | 4 | 4 | 4 |  |  |  |  |  |  |  |  |  |  |  |  |  |  |
| 1313 | 2 | 4 | 2 | 4 | 2 | 4 | 6 | 6 | 7 | 9 | 9 | 10 | 8 | 9 | 9 |  | 12 | 12 |
| 1315 | 3 | 4 | 2 | 4 |  |  |  |  |  |  |  |  |  |  |  |  |  |  |
| 1379 | 2 | 4 | 1 | 4 | 2 | 4 |  | 3 | 3 | 10 | 6 |  | 6 | 6 | 6 |  | 12 |  |
| 1384 | 8 | 4 |  |  |  |  |  |  |  |  |  |  |  |  |  |  |  |  |
| 1394 | 2 | 4 | 2 | 4 | 2 | 4 | 9 | 7 |  | 10 | 8 | 11 | 6 | 10 | 8 | 8 |  |  |
| 1479 | 2 | 4 |  |  | 2 | 4 | 4 | 4 | 5 | 5 | 6 | 6 | 6 | 6 | 6 | 9 | 9 | 12 |
| 1481 | 4 | 4 |  |  |  |  |  |  |  |  |  |  |  |  |  |  |  |  |
| 1557 | 2 | 4 | 2 | 4 | 2 | 4 | 4 | 4 | 6 | 9 | 6 |  | 6 | 6 | 6 | 7 | 12 | 11 |
| 1629 | 16 | 4 | 5 | 4 | 8 | 4 |  | 6 | 6 | 7 | 6 |  | 6 | 6 | 6 | 10 |  |  |
| 1649 | 3 | 4 |  |  | 2 | 4 | 4 | 4 | 5 | 6 | 5 | 8 | 5 | 5 | 5 | 11 | 12 |  |
| 1677 | 3 | 6 | 4 | 6 | 3 | 0 |  | 5 | 6 | 7 | 6 |  | 9 | 5 | 6 | 10 | 11 | 11 |
| 1711 | 4 | 5 | 3 | 3 | 4 | 4 |  |  | 4 | 6 | 6 | 9 | 6 | 5 | 5 | 8 | 10 |  |
| 1836 | 4 | 4 | 4 | 4 |  |  |  |  |  |  |  |  |  |  |  |  |  |  |
| 1905 | 4 | 4 | 4 | 6 | 2 | 2 | 4 | 4 | 5 | 9 | 6 |  | 4 | 4 |  | 12 | 12 |  |
| 1981 | 4 | 4 | 4 | 4 |  |  |  |  |  |  |  |  |  |  |  |  |  |  |
| 1998 | 4 | 4 | 4 | 4 | 2 | 4 |  |  | 5 | 11 | 12 | 16 | 6 | 6 | 11 |  | 12 | 18 |
| 2033 | 3 | 4 | 2 | 4 |  |  |  |  |  |  |  |  |  |  |  |  |  |  |
| 2034 | 1 | 3 | 3 | 5 |  |  |  |  |  |  |  |  |  |  |  |  |  |  |
| 2043 | 4 | 4 | 4 | 4 |  |  |  |  |  |  |  |  |  |  |  |  |  |  |
| 2103 | 4 | 4 |  |  |  |  |  |  |  |  |  |  |  |  |  |  |  |  |
| 2161 | 1 | 4 | 1 | 4 |  |  |  |  |  |  |  |  |  |  |  |  |  |  |
| 2170 | 4 | 4 | 4 | 4 | 4 | 4 |  |  | 6 | 9 | 9 |  | 8 | 9 | 9 | 9 | 12 |  |
| 2207 | 2 | 4 | 2 | 4 | 2 | 4 | 6 | 6 | 6 | 8 | 7 |  | 6 | 8 | 8 | 8 | 12 | 10 |
| 2411 | 4 | 4 |  |  |  |  |  |  |  |  |  |  |  |  |  |  |  |  |
| 2503 | 2 | 4 | 4 | 4 | 4 | 4 | 4 | 4 | 6 | 8 | 10 |  | 8 | 8 | 8 | 12 | 12 | 12 |
| 2591 | 1 | 4 | 4 | 4 | 4 | 4 | 10 |  | 7 | 7 | 7 | 9 | 6 | 7 | 7 | 8 | 10 |  |
| 2629 | 3 | 5 | 2 | 4 | 3 | 3 | 3 | 3 | 5 | 10 | 10 | 10 | 4 | 4 | 10 |  | 11 | 11 |
| 2652 | 2 | 4 | 2 | 4 | 1 | 4 | 4 | 4 | 5 | 8 | 6 | 8 | 6 | 6 | 10 | 12 | 10 | 12 |
| 20080 | 8 | 4 |  |  |  |  |  |  |  |  |  |  |  |  |  |  |  |  |
| 20084 | 4 | 4 | 4 |  |  |  |  |  |  |  |  |  |  |  |  |  |  |  |
| 200 | 3 | 5 | 1 | 4 | 1 | 4 |  |  | 4 | 9 | 4 |  | 6 | 5 | 5 |  |  |  |
| 1135 | 2 | 2 | 2 | 3 | 2 | 8 | 4 | 4 | 4 | 6 | 7 | 7 | 7 | 4 | 8 | 8 | 11 | 10 |
| 1202 | 1 | 1 | 4 | 4 | 4 | 8 |  | 6 | 7 | 8 | 8 | 10 | 7 | 8 | 8 | 10 |  | 10 |
| 1345 | 4 | 4 | 2 | 4 | 2 | 5 | 5 | 5 | 7 | 7 | 6 |  | 9 | 6 | 6 | 13 | 12 | 12 |
| 1367 | 4 | 6 | 4 | 3 | 3 | 4 | 6 |  | 6 | 8 | 10 |  | 8 | 7 | 10 |  |  |  |
| 1647 | 3 | 3 | 4 | 4 | 4 | 6 | 6 | 6 |  | 9 | 9 |  | 7 | 6 | 6 | 12 | 12 | 10 |
| 1855 | 2 | 4 | 3 | 3 | 4 | 6 |  |  | 4 | 5 | 6 | 10 | 6 | 6 | 7 | 10 | 11 | 10 |
| 2061 | 3 | 3 | 3 | 4 | 2 | 6 |  | 6 | 6 | 9 | 7 |  | 7 | 9 | 9 | 12 | 12 |  |
| 2065 | 3 | 3 | 2 | 4 | 2 | 6 | 12 | 6 | 6 | 12 | 6 |  | 8 | 6 | 8 | 11 | 10 | 12 |
| 2256 |  | 4 | 1 | 4 | 1 | 5 | 5 | 5 | 9 | 7 | 9 | 10 | 6 | 8 | 8 | 10 | 11 |  |
| 2451 | 2 | 4 | 3 | 3 | 2 | 6 |  | 6 | 6 | 7 | 7 | 10 | 4 | 7 | 7 | 7 | 10 | 10 |
| 129 | 2 | 4 | 3 | 5 |  |  |  |  |  |  |  |  |  |  |  |  |  |  |
| 1331 | 4 | 5 | 5 | 4 |  |  |  |  |  |  |  |  |  |  |  |  |  |  |
| 1335 | 2 | 4 | 2 | 5 |  |  |  |  |  |  |  |  |  |  |  |  |  |  |
| 1715 | 4 | 4 | 3 | 5 |  |  |  |  |  |  |  |  |  |  |  |  |  |  |
| 2049 | 3 | 5 | 2 | 4 |  |  |  |  |  |  |  |  |  |  |  |  |  |  |
| 2621 | 2 | 6 | 2 | 3 |  |  |  |  |  |  |  |  |  |  |  |  |  |  |
| 217 | 2 | 6 | 4 | 4 | 1 | 4 |  |  | 6 | 10 | 12 |  | 6 | 6 | 10 | 12 | 12 |  |
| 305 | 2 | 4 | 2 | 6 | 2 | 4 | 5 |  | 5 | 68 | 6 |  | 6 | 5 | 6 | 6 | 10 | 12 |
| 1143 | 4 | 4 | 4 | 4 | 4 | 6 | 6 | 6 | 6 | 8 | 6 | 10 | 6 | 7 | 9 | 9 |  |  |
| 1414 | 2 | 5 | 3 | 5 | 2 | 4 | 4 | 4 | 6 | 8 | 5 | 11 | 4 | 5 | 6 | 10 | 12 | 10 |
| 1473 | 4 | 6 | 4 | 4 | 2 | 4 | 8 | 8 | 8 | 10 | 9 |  | 10 | 9 | 9 |  |  |  |
| 1630 | 3 | 4 | 3 | 5 | 3 | 5 |  |  |  |  |  |  |  |  |  |  | 12 |  |
| 1704 | 4 | 6 | 2 | 4 | 2 | 4 |  | 5 | 6 | 8 | 8 | 10 | 8 | 6 | 6 | 10 | 12 |  |
| 2227 | 2 | 4 | 3 | 5 | 3 | 5 | 12 |  | 6 | 10 | 11 |  | 6 | 6 | 12 |  | 7 |  |
| 20074 | 3 | 3 | 4 | 6 | 3 | 5 |  |  | 9 | 9 | 9 |  | 9 | 9 | 9 | 9 | 9 |  |
| 138 | 3 | 4 | 2 | 5 | 2 | 6 | 4 | 4 | 5 | 6 | 6 | 10 | 10 | 6 |  | 10 | 10 | 10 |
| 228 | 3 | 5 | 1 | 8 | 2 | 2 | 11 |  | 4 | 6 | 6 | 9 | 8 | 7 | 6 | 11 | 11 |  |
| 1326 | 3 | 5 | 1 | 4 | 3 | 6 |  | 8 | 11 | 11 | 8 |  | 8 |  | 9 | 10 |  |  |
| 1361 | 4 | 7 | 4 | 3 | 8 | 5 | 6 | 6 |  | 7 | 7 | 9 | 6 |  | 7 |  |  |  |
| 1395 | 4 | 8 | 5 | 2 |  |  |  |  |  |  |  |  |  |  |  |  |  |  |
| 1486 | 4 | 6 | 4 | 4 |  |  |  |  |  |  |  |  |  |  |  |  |  |  |
| 1497 | 1 | 3 | 1 | 3 | 1 | 9 |  |  | 6 | 10 | 6 |  | 6 | 6 | 9 | 8 | 11 |  |
| 1566 | 3 | 4 |  |  | 2 | 6 | 6 | 6 | 6 | 6 | 6 |  | 6 | 6 | 6 | 6 | 12 |  |
| 1648 | 4 | 5 |  |  |  |  |  |  |  |  |  |  |  |  |  |  |  |  |
| 1842 | 3 | 6 | 3 | 4 | 2 | 5 | 4 |  | 5 | 7 | 9 |  | 4 | 4 | 10 | 10 | 11 |  |
| 1849 | 4 | 4 | 4 | 5 | 3 | 6 | 8 | 8 | 6 | 8 | 6 | 12 | 10 | 6 | 6 | 8 | 10 | 14 |
| 1902 | 4 | 6 | 4 | 4 |  |  |  |  |  |  |  |  |  |  |  |  |  |  |
| 1929 | 9 | 5 |  |  |  |  |  |  |  |  |  |  |  |  |  |  |  |  |
| 1939 | 2 | 5 |  |  |  |  |  |  |  |  |  |  |  |  |  |  |  |  |
| 1944 | 1 | 4 | 3 | 6 | 4 | 5 |  |  |  | 9 | 9 | 6 | 6 | 11 | 6 | 9 | 11 |  |
| 1951 | 1 | 6 | 1 | 4 | 1 | 5 |  | 6 | 6 | 9 | 8 | 11 | 9 | 8 | 9 | 12 | 11 |  |
| 1984 | 4 | 4 |  |  | 5 | 6 | 3 | 4 | 6 | 7 | 8 | 12 | 11 | 4 | 6 | 10 | 12 |  |
| 2038 | 1 | 5 |  |  |  |  |  |  |  |  |  |  |  |  |  |  |  |  |
| 2050 | 3 | 5 |  |  |  |  |  |  |  |  |  |  |  |  |  |  |  |  |
| 2051 | 2 | 6 | 2 | 4 |  |  |  |  |  |  |  |  |  |  |  |  |  |  |
| 2088 | 6 | 5 |  |  |  |  |  |  |  |  |  |  |  |  |  |  |  |  |
| 2542 | 3 | 6 | 2 | 4 |  |  |  |  |  |  |  |  |  |  |  |  |  |  |
| 2600 | 2 | 7 | 1 | 3 | 3 | 5 | 3 | 3 | 4 | 7 | 5 |  | 4 | 5 | 5 | 5 | 11 | 10 |
| 20077 | 2 | 5 | 2 | 5 |  |  |  |  |  |  |  |  |  |  |  |  |  |  |
| 1505 | 3 | 6 | 2 | 6 | 2 | 4 |  |  | 6 | 9 | 9 |  | 6 | 6 | 6 | 6 |  |  |
| 1628 | 4 | 8 | 4 | 6 | 8 | 2 | 4 | 4 | 5 | 8 | 4 |  | 4 | 6 | 7 | 11 |  | 11 |
| 1642 | 2 | 4 | 2 | 8 | 4 | 4 |  | 4 | 5 | 6 | 6 | 9 | 6 | 7 | 6 | 12 | 12 | 12 |
| 1643 | 3 | 6 | 2 | 4 | 4 | 6 |  | 7 | 7 | 8 | 8 |  | 8 | 9 | 8 | 11 | 11 | 9 |
| 1899 | 2 | 4 | 2 | 4 | 4 | 8 |  | 6 | 6 | 6 | 6 |  | 6 | 6 | 6 | 6 | 11 |  |
| 1977 | 2 | 4 | 2 | 8 | 2 | 4 | 6 | 6 | 8 | 8 | 6 |  | 6 | 8 |  |  |  |  |
| 2140 | 2 | 4 | 3 | 4 | 4 | 8 |  | 4 | 6 | 6 | 36 | 11 | 6 | 6 | 6 | 10 | 12 | 12 |
| 2206 | 1 | 2 | 1 | 6 | 2 | 8 |  |  | 5 | 5 |  |  | 6 | 4 | 4 |  |  |  |
| 780 | 2 | 3 |  |  | 2 | 8 | 6 | 6 | 6 | 6 | 6 | 10 | 8 | 8 | 8 | 10 | 12 |  |
| 1337 | 1 | 8 | 2 | 3 |  |  |  |  |  |  |  |  |  |  |  |  |  |  |
| 1824 | 2 | 7 |  |  | 2 | 4 | 6 | 6 | 7 | 10 | 6 | 9 | 6 | 6 | 6 | 8 | 12 | 70 |
| 1886 | 3 | 5 | 3 | 6 |  |  |  |  |  |  |  |  |  |  |  |  |  |  |
| 1612 | 4 | 4 | 5 | 8 | 3 | 5 |  | 4 | 6 | 8 | 6 |  | 6 | 6 | 6 | 6 |  |  |
| 1616 | 4 | 5 | 1 | 8 | 2 | 4 | 7 |  | 8 | 12 | 7 | 12 | 8 | 7 | 10 | 12 |  |  |
| 1909 | 3 | 5 | 3 | 7 | 4 | 5 | 5 | 6 | 7 | 5 | 6 | 12 | 5 | 7 | 6 | 8 | 12 | 12 |
| 104 | 5 | 6 |  |  |  |  |  |  |  |  |  |  |  |  |  |  |  |  |
| 105 | 4 | 6 | 4 | 6 |  |  |  |  |  |  |  |  |  |  |  |  |  |  |
| 123 | 3 | 4 |  |  | 5 | 8 |  |  | 6 | 8 | 5 |  | 78 | 8 | 8 | 11 | 11 |  |
| 190 | 3 | 6 |  |  |  |  |  |  |  |  |  |  |  |  |  |  |  |  |
| 1261 | 3 | 6 |  |  |  |  |  |  |  |  |  |  |  |  |  |  |  |  |
| 1332 | 4 | 6 |  |  |  |  |  |  |  |  |  |  |  |  |  |  |  |  |
| 1386 | 4 | 6 |  |  |  |  |  |  |  |  |  |  |  |  |  |  |  |  |
| 1389 | 4 | 6 | 4 | 6 | 4 | 6 | 12 |  | 5 | 8 | 11 |  | 9 | 7 | 11 | 11 |  |  |
| 1419 | 2 | 8 | 2 | 8 | 2 | 2 | 6 | 6 | 6 | 6 | 6 | 6 | 6 | 6 | 6 | 6 | 5 | 6 |
| 1537 | 2 | 6 |  |  |  |  |  |  |  |  |  |  |  |  |  |  |  |  |
| 1554 | 2 | 4 | 4 | 4 | 4 | 10 |  | 6 | 7 | 10 | 8 | 11 | 11 | 8 | 12 | 10 |  |  |
| 1695 | 8 | 2 | 10 | 10 |  |  |  |  |  |  |  |  |  |  |  |  |  |  |
| 1857 | 1 | 6 |  |  |  |  |  |  |  |  |  |  |  |  |  |  |  |  |
| 1894 | 4 | 4 | 4 | 8 |  |  |  |  |  |  |  |  |  |  |  |  |  |  |
| 1950 | 5 | 5 | 5 | 5 | 8 | 8 | 6 | 6 | 8 | 9 | 8 |  | 7 | 8 | 8 |  |  |  |
| 2042 | 4 |  | 4 | 6 |  |  |  |  |  |  |  |  |  |  |  |  |  |  |
| 2057 | 4 | 6 |  |  |  |  |  |  |  |  |  |  |  |  |  |  |  |  |
| 2567 | 4 | 6 | 3 |  |  |  |  |  |  |  |  |  |  |  |  |  |  |  |
| 2627 | 6 | 6 |  |  |  |  |  |  |  |  |  |  |  |  |  |  |  |  |
| 20083 | 4 | 6 |  |  |  |  |  |  |  |  |  |  |  |  |  |  |  |  |
| 139 | 4 | 8 | 4 | 3 | 4 | 8 | 4 | 4 |  | 9 | 7 |  | 7 |  | 7 | 12 | 12 |  |
| 1144 | 4 | 6 | 3 | 8 | 3 | 5 |  | 6 | 8 | 8 | 6 | 8 | 6 | 6 | 6 | 10 | 12 | 12 |
| 1217 | 4 | 5 | 4 | 6 | 4 | 8 |  |  | 9 | 10 | 10 |  | 8 |  | 8 | 11 | 11 |  |
| 1307 | 5 | 5 | 5 | 8 | 4 | 6 | 6 |  | 8 | 7 | 6 | 8 | 8 | 6 | 6 | 10 | 10 |  |
| 1533 | 4 | 4 | 8 | 7 | 4 | 8 | 4 | 5 | 5 | 8 | 6 | 9 | 5 | 6 | 8 |  | 12 |  |
| 1542 | 2 | 5 | 2 | 4 | 4 | 10 | 4 | 5 | 6 | 5 | 5 |  | 6 | 6 | 6 | 8 | 12 | 12 |
| 2603 | 6 | 6 | 4 | 7 | 4 | 6 | 6 | 6 |  | 11 | 9 | 11 | 8 | 8 | 11 |  |  |  |
| 1852 | 3 | 3 | 3 | 10 |  |  |  |  |  |  |  |  |  |  |  |  |  |  |
| 1882 | 2 | 8 |  |  | 5 | 5 |  | 6 | 6 | 10 | 10 | 10 | 6 | 5 |  |  |  |  |
| 2446 | 2 | 8 | 2 | 5 |  |  |  |  |  |  |  |  |  |  |  |  |  |  |
| 1318 | 1 | 8 | 2 | 6 | 1 | 6 | 6 | 7 | 8 | 8 | 8 |  | 6 | 7 | 8 | 7 | 12 | 8 |
| 1485 | 1 | 8 | 1 | 6 | 4 | 6 | 4 | 4 | 5 | 10 | 6 |  | 6 |  | 6 | 12 | 12 | 12 |
| 1614 | 2 | 4 | 4 | 8 | 3 | 8 | 5 | 5 | 4 | 8 | 10 |  |  | 9 | 8 | 8 | 11 |  |
| 1821 | 4 | 8 | 4 | 8 | 3 | 4 | 10 | 5 | 5 | 10 | 5 | 7 | 5 | 6 | 5 | 5 |  | 7 |
| 1863 | 4 | 4 | 3 | 8 | 3 | 8 |  |  | 6 | 10 | 10 |  | 8 | 10 | 10 | 12 | 12 | 12 |
| 1904 | 1 | 6 | 1 | 6 | 3 | 8 | 5 |  | 6 | 8 | 8 | 10 | 6 | 8 |  | 12 |  |  |
| 1956 | 3 | 5 | 5 | 5 | 5 | 10 |  |  | 5 | 9 | 6 | 10 | 6 | 6 | 9 | 11 | 9 | 10 |
| 2168 | 2 | 6 | 2 | 6 | 2 | 8 | 11 | 5 | 8 | 9 | 7 | 11 | 6 | 5 | 5 |  |  |  |
| 2203 | 4 | 4 | 4 | 6 | 4 | 10 |  |  | 6 | 8 | 9 |  | 6 | 9 | 9 | 9 |  |  |
| 128 | 3 | 6 |  |  | 5 | 8 | 6 | 6 | 8 | 10 | 10 |  | 10 | 8 | 11 | 12 | 11 |  |
| 324 | 4 | 4 | 4 | 10 |  |  |  |  |  |  |  |  |  |  |  |  |  |  |
| 1235 | 2 | 5 | 2 | 8 | 2 | 8 | 10 | 6 | 6 | 10 | 11 | 11 | 10 |  | 10 | 10 | 11 |  |
| 1382 | 2 | 4 | 4 | 10 |  |  |  |  |  |  |  |  |  |  |  |  |  |  |
| 1539 | 4 | 8 | 5 | 5 | 5 | 8 | 4 | 5 | 5 | 5 | 8 | 8 | 6 | 8 | 8 |  | 8 | 8 |
| 1555 | 2 | 6 | 2 | 8 |  |  |  |  |  |  |  |  |  |  |  |  |  |  |
| 1687 | 8 | 4 | 9 | 9 | 6 | 8 | 4 | 4 | 5 | 6 | 6 | 6 | 6 | 6 | 6 | 6 | 12 |  |
| 1710 | 2 | 10 | 2 | 4 |  |  |  |  |  |  |  |  |  |  |  |  |  |  |
| 1846 | 3 | 5 | 4 | 9 |  |  |  |  |  |  |  |  |  |  |  |  |  |  |
| 2104 | 4 | 7 |  |  |  |  |  |  |  |  |  |  |  |  |  |  |  |  |
| 2150 | 3 | 5 | 4 | 8 | 4 | 8 |  |  | 6 | 7 | 6 | 10 | 7 | 7 | 7 | 11 | 11 | 11 |
| 2243 |  |  | 4 | 6 | 4 | 8 |  | 5 | 7 | 6 | 6 | 8 | 6 | 6 | 6 | 0 | 11 |  |
| 2565 | 3 | 3 | 2 | 6 | 2 | 12 |  | 4 | 6 | 6 | 6 | 10 | 9 | 6 | 6 | 11 | 12 | 12 |
| 2579 | 5 | 7 |  |  |  |  |  |  |  |  |  |  |  |  |  |  |  |  |
| 2624 | 4 | 4 | 4 | 10 |  |  |  |  |  |  |  |  |  |  |  |  |  |  |
| 1284 | 4 | 6 | 6 | 8 | 6 | 8 | 5 | 5 | 4 | 6 | 8 | 8 | 4 | 4 | 8 | 8 | 12 | 8 |
| 1342 | 4 | 8 | 4 | 8 | 3 | 6 | 5 | 5 | 4 | 7 | 7 | 12 | 5 | 6 | 10 | 7 | 11 |  |
| 1392 | 4 | 7 | 4 | 8 | 3 | 7 | 6 | 6 | 6 | 9 | 8 |  | 6 | 6 | 7 | 9 |  | 11 |
| 1521 | 5 | 8 | 4 | 8 | 4 | 6 |  | 4 | 6 | 10 | 6 |  | 6 | 6 | 6 | 10 | 12 | 10 |
| 1639 | 2 | 6 | 2 | 8 | 2 | 8 |  | 5 | 6 | 11 | 10 |  | 8 | 10 | 10 | 10 | 11 | 12 |
| 2391 | 2 | 6 | 3 | 8 | 2 | 8 | 6 |  | 7 | 8 | 8 |  | 6 | 9 | 8 | 11 |  |  |
| 2632 | 1 | 8 | 2 | 4 | 1 | 10 | 6 | 6 | 6 | 8 | 6 | 11 | 6 | 6 | 6 | 6 |  |  |
| 1138 | 4 | 8 | 4 | 7 |  |  |  |  |  |  |  |  |  |  |  |  |  |  |
| 1354 | 4 | 7 | 4 | 8 |  |  |  |  |  |  |  |  |  |  |  |  |  |  |
| 1565 | 3 | 7 | 4 | 8 |  |  |  |  |  |  |  |  |  |  |  |  |  |  |
| 1895 | 2 | 7 | 3 | 8 |  |  |  |  |  |  |  |  |  |  |  |  |  |  |
| 1376 | 3 | 6 | 3 | 7 | 3 | 10 | 4 | 4 | 5 | 10 | 11 |  | 8 | 4 | 11 |  | 11 |  |
| 1995 | 4 | 6 | 3 | 5 | 4 | 12 | 3 | 13 | 3 | 7 | 11 |  | 6 | 4 | 8 | 11 | 11 | 12 |
| 2213 | 2 | 4 | 2 | 4 | 4 | 15 |  |  | 6 | 8 | 8 |  | 10 | 6 | 7 |  | 11 |  |
| 2564 | 5 | 7 | 4 | 8 | 4 | 8 | 13 | 5 | 6 | 6 | 8 | 8 | 5 | 5 | 5 | 8 | 12 |  |
| 136 | 2 | 8 | 2 | 8 |  |  |  |  |  |  |  |  |  |  |  |  |  |  |
| 932 | 4 | 8 | 4 | 8 | 4 | 8 |  |  | 4 | 9 | 4 | 11 | 4 | 4 | 4 | 10 | 10 |  |
| 1314 | 3 | 8 | 3 | 8 | 3 | 8 |  | 4 | 6 | 8 | 8 | 8 | 8 | 4 | 6 | 7 | 9 |  |
| 1339 | 4 | 8 |  |  |  |  |  |  |  |  |  |  |  |  |  |  |  |  |
| 1366 | 4 | 8 |  |  | 2 | 8 | 12 | 4 | 4 | 6 | 6 | 10 | 6 | 6 | 12 | 12 | 12 |  |
| 1455 | 4 | 12 | 3 | 4 | 4 | 8 | 6 | 6 | 6 | 8 | 9 |  | 11 | 8 | 8 | 8 | 7 |  |
| 1461 | 5 | 10 | 4 | 6 | 4 | 8 | 4 |  | 6 | 8 | 4 |  | 6 | 4 | 6 | 10 | 10 |  |
| 1491 | 4 | 8 |  |  |  |  |  |  |  |  |  |  |  |  |  |  |  |  |
| 1506 | 4 | 8 |  |  |  |  |  |  |  |  |  |  |  |  |  |  |  |  |
| 1548 | 4 | 8 | 4 | 8 | 4 | 8 |  | 5 | 8 | 7 | 6 | 10 | 6 | 6 | 10 | 10 |  |  |
| 1551 | 4 | 8 |  |  |  |  |  |  |  |  |  |  |  |  |  |  |  |  |
| 1635 | 4 | 10 | 3 | 6 | 5 | 8 | 4 | 6 | 6 | 10 | 10 | 8 | 10 | 6 | 6 | 12 | 12 |  |
| 1658 | 4 | 8 |  |  |  |  |  |  |  |  |  |  |  |  |  |  |  |  |
| 1725 | 4 | 8 | 4 | 8 |  |  |  |  |  |  |  |  |  |  |  |  |  |  |
| 1768 | 3 | 4 | 3 | 12 |  |  |  |  |  |  |  |  |  |  |  |  |  |  |
| 1835 |  |  |  |  | 4 | 8 |  | 5 |  | 7 | 7 |  | 6 |  | 8 |  | 10 |  |
| 1952 | 4 | 8 |  |  |  |  |  |  |  |  |  |  |  |  |  |  |  |  |
| 1979 | 4 | 8 |  |  |  |  |  |  |  |  |  |  |  |  |  |  |  |  |
| 1987 | 3 | 7 | 4 | 9 |  |  |  |  |  |  |  |  |  |  |  |  |  |  |
| 2142 | 4 | 8 |  |  |  |  |  |  |  |  |  |  |  |  |  |  |  |  |
| 2214 | 2 | 8 |  |  |  |  |  |  |  |  |  |  |  |  |  |  |  |  |
| 2229 | 4 | 8 |  |  | 2 | 8 | 64 | 4 | 5 | 6 | 6 |  | 7 | 6 | 6 | 9 | 11 |  |
| 2244 | 4 | 8 |  |  |  |  |  |  |  |  |  |  |  |  |  |  |  |  |
| 2254 | 4 | 8 |  |  |  |  |  |  |  |  |  |  |  |  |  |  |  |  |
| 2423 | 3 | 8 |  |  |  |  |  |  |  |  |  |  |  |  |  |  |  |  |
| 2436 | 4 | 8 |  |  |  |  |  |  |  |  |  |  |  |  |  |  |  |  |
| 2547 | 3 | 8 |  |  |  |  |  |  |  |  |  |  |  |  |  |  |  |  |
| 2570 | 4 | 4 | 3 | 12 |  |  |  |  |  |  |  |  |  |  |  |  |  |  |
| 2592 | 4 | 8 | 4 | 8 | 8 | 8 |  |  | 5 | 8 | 6 | 8 | 6 | 6 | 6 | 10 | 11 |  |
| 20072 | 4 | 8 |  |  |  |  |  |  |  |  |  |  |  |  |  |  |  |  |
| 195 | 3 | 5 | 4 | 12 | 4 | 8 | 5 | 5 | 6 | 10 | 10 |  | 12 | 6 | 8 | 8 | 12 |  |
| 2083 | 4 | 9 | 5 | 8 | 4 | 8 | 6 | 6 | 6 | 7 | 7 | 8 | 5 |  | 7 | 11 |  |  |
| 1714 | 6 | 15 | 8 |  | 8 | 2 |  | 3 | 5 | 8 | 5 | 11 | 5 | 5 | 5 | 12 | 8 | 12 |
| 1230 | 1 | 8 | 2 | 10 | 3 | 8 |  |  |  | 10 | 10 | 11 | 9 |  | 10 |  | 12 |  |
| 1484 | 3 | 8 | 3 | 10 | 1 | 8 |  | 4 | 8 | 11 | 6 |  | 6 | 10 | 6 | 6 | 12 |  |
| 2062 | 2 | 4 | 2 | 10 | 2 | 12 | 4 | 4 | 5 | 6 | 6 | 10 | 4 |  | 6 | 6 |  |  |
| 147 | 5 | 8 |  |  | 4 | 10 | 6 | 6 | 6 | 10 | 10 |  | 7 | 6 | 10 | 10 | 12 |  |
| 311 | 3 | 6 | 6 | 12 |  |  |  |  |  |  |  |  |  |  |  |  |  |  |
| 1319 | 6 | 8 |  |  | 6 | 10 | 5 | 5 | 5 | 8 | 7 |  | 8 | 8 | 11 |  | 11 |  |
| 1353 | 2 | 12 | 2 | 6 |  |  |  |  |  |  |  |  |  |  |  |  |  |  |
| 1471 | 3 | 6 |  |  | 4 | 12 | 10 | 4 |  | 6 | 6 | 7 | 6 | 6 | 6 | 12 | 12 |  |
| 2225 |  |  | 4 | 10 | 4 | 8 |  | 4 | 4 | 9 | 6 | 12 | 6 | 6 | 6 | 6 | 10 |  |
| 1226 | 3 | 6 | 4 | 12 | 3 | 10 | 4 | 4 | 5 | 7 | 6 |  | 6 | 5 | 11 |  | 11 | 10 |
| 1321 | 4 | 8 | 4 | 8 | 3 | 12 |  | 5 | 5 | 10 | 5 |  | 4 | 5 | 5 | 5 | 12 | 6 |
| 1373 | 5 | 12 | 4 | 8 | 4 | 8 | 6 | 5 | 5 | 5 | 6 | 7 | 6 | 6 | 6 | 8 | 11 |  |
| 1527 | 4 | 8 | 4 | 8 | 6 | 12 | 5 | 5 | 7 | 7 | 5 |  | 5 | 6 | 5 |  | 11 |  |
| 1528 | 3 | 7 | 3 | 6 | 3 | 15 |  | 4 | 4 | 9 | 9 |  | 5 | 4 | 9 | 9 | 9 | 9 |
| 307 | 2 | 10 |  |  |  |  |  |  |  |  |  |  |  |  |  |  |  |  |
| 317 | 4 | 8 | 4 | 14 | 4 | 8 |  | 4 | 5 | 9 | 6 |  | 6 | 6 | 8 |  | 12 |  |
| 1211 | 4 | 12 | 4 | 8 | 8 | 10 |  | 3 | 3 | 8 | 6 |  | 6 | 3 | 3 | 12 | 10 |  |
| 1368 | 6 | 10 |  |  |  |  |  |  |  |  |  |  |  |  |  |  |  |  |
| 1383 | 5 | 10 |  |  |  |  |  |  |  |  |  |  |  |  |  |  |  |  |
| 1437 | 3 | 10 |  |  |  |  |  |  |  |  |  |  |  |  |  |  |  |  |
| 1514 | 5 | 10 | 6 | 10 | 6 | 10 | 4 | 4 | 7 | 8 | 7 | 10 | 6 | 8 | 6 | 8 | 11 |  |
| 1531 | 3 | 10 |  |  |  |  |  |  |  |  |  |  |  |  |  |  |  |  |
| 1561 | 6 | 10 |  |  |  |  |  |  |  |  |  |  |  |  |  |  |  |  |
| 1625 | 5 | 10 | 8 |  |  |  |  |  |  |  |  |  |  |  |  |  |  |  |
| 1946 | 4 | 10 |  |  |  |  |  |  |  |  |  |  |  |  |  |  |  |  |
| 2594 | 4 | 10 |  |  |  |  |  |  |  |  |  |  |  |  |  |  |  |  |
| 1563 | 4 | 15 |  | 10 | 3 | 6 |  |  | 4 | 9 | 10 | 17 | 11 | 4 | 6 |  | 10 | 11 |
| 2212 | 4 | 8 | 8 | 8 | 4 | 15 | 4 | 4 | 5 | 6 |  |  | 6 | 4 | 5 | 8 | 12 |  |
| 1218 | 8 | 12 | 4 | 8 | 6 | 12 | 4 | 4 | 5 | 8 | 8 |  | 5 | 4 | 4 | 8 | 11 | 10 |
| 2558 | 5 | 10 | 5 | 10 | 4 | 12 |  | 6 | 6 | 6 | 8 | 8 | 6 |  | 8 | 8 | 12 | 12 |
| 931 | 5 | 10 | 5 | 12 |  |  |  |  |  |  |  |  |  |  |  |  |  |  |
| 1147 | 8 | 12 | 4 | 10 |  |  |  |  |  |  |  |  |  |  |  |  |  |  |
| 1477 | 3 | 8 | 4 | 10 | 3 | 15 | 4 | 4 | 5 | 8 | 8 | 8 | 8 | 6 | 6 | 0 | 0 | 0 |
| 1532 | 4 | 12 | 4 | 10 |  |  |  |  |  |  |  |  |  |  |  |  |  |  |
| 1543 | 12 | 9 | 7 | 12 | 10 | 12 | 11 | 6 | 7 | 10 | 9 |  | 10 | 7 | 9 | 11 | 11 |  |
| 1559 | 4 | 12 | 4 | 10 |  |  |  |  |  |  |  |  |  |  |  |  |  |  |
| 213 | 4 | 8 | 4 | 15 |  |  |  |  |  |  |  |  |  |  |  |  |  |  |
| 1515 | 2 | 13 | 2 | 12 | 3 | 10 |  |  |  |  |  |  |  |  |  |  |  |  |
| 306 | 6 | 12 |  |  |  |  |  |  |  |  |  |  |  |  |  |  |  |  |
| 467 | 4 | 16 | 4 | 12 | 4 | 8 | 4 | 4 | 6 | 8 | 9 | 12 | 9 | 6 | 10 | 10 | 10 | 11 |
| 1301 | 4 | 12 |  |  |  |  |  |  |  |  |  |  |  |  |  |  |  |  |
| 1370 | 3 | 12 |  |  |  |  |  |  |  |  |  |  |  |  |  |  |  |  |
| 1478 | 4 | 12 | 4 | 10 | 4 | 14 | 5 | 5 | 3 | 9 | 6 |  | 4 | 4 | 6 |  | 12 |  |
| 2101 | 4 | 8 | 4 | 8 | 4 | 20 |  | 7 |  |  | 8 |  | 8 | 8 |  | 8 |  |  |
| 20075 | 3 | 8 | 3 | 8 | 3 | 20 |  |  | 4 | 6 | 6 |  | 10 | 6 | 9 |  | 10 |  |
| 1385 | 4 | 12 | 4 | 16 | 2 | 9 | 4 | 4 | 5 | 8 | 6 | 9 | 4 | 6 | 6 | 12 | 12 | 12 |
| 940 | 5 | 10 | 4 | 15 |  |  |  |  |  |  |  |  |  |  |  |  |  |  |
| 2091 | 4 | 8 | 3 | 12 | 3 | 18 | 4 | 4 | 6 | 8 | 6 | 9 | 5 | 6 | 5 | 6 | 12 | 12 |
| 2622 | 6 | 10 | 4 | 16 |  |  |  |  |  |  |  |  |  |  |  |  |  |  |
| 1834 | 12 | 15 | 10 | 10 | 8 | 15 |  |  |  | 6 |  |  |  | 3 | 4 | 12 | 7 |  |
| 1611 | 4 | 10 | 3 | 11 | 4 | 20 | 3 | 3 | 4 | 5 |  |  |  | 4 |  |  | 11 |  |
| 148 |  |  | 4 | 8 | 4 | 20 |  | 5 | 7 | 9 | 6 |  | 7 | 7 | 7 | 9 |  |  |
| 930 | 4 | 8 | 8 | 15 | 4 | 20 |  | 6 | 6 | 8 | 8 |  | 6 |  | 8 | 1 | 18 |  |
| 227 | 5 | 10 |  |  | 5 | 20 | 5 | 4 | 5 | 7 | 7 | 0 | 10 | 9 | 9 | 9 | 10 |  |
| 922 | 3 | 15 |  |  |  |  |  |  |  |  |  |  |  |  |  |  |  |  |
| 1329 | 3 | 20 | 3 | 8 | 2 | 20 |  | 4 | 5 | 4 | 6 |  | 5 | 6 | 6 | 8 | 10 |  |
| 1936 | 4 | 20 | 4 | 16 | 5 | 14 | 6 | 6 | 9 | 8 | 10 |  | 6 | 9 | 10 |  |  |  |
| 1985 | 8 | 12 | 2 | 8 | 4 | 30 | 10 | 6 | 6 | 10 | 8 | 10 | 7 | 8 | 8 | 11 | 12 | 13 |
| 1150 | 3 | 4 |  |  | 4 | 30 | 6 | 6 | 7 | 8 | 7 |  | 7 | 7 | 1 | 10 |  |  |
| 1380 | 15 | 20 | 8 | 14 |  |  |  |  |  |  |  |  |  |  |  |  |  |  |
| 2236 | 4 | 12 | 2 | 20 | 4 | 20 | 12 | 6 | 6 | 8 | 8 | 8 | 8 | 6 | 8 | 12 | 12 | 12 |
| 2635 | 2 | 18 |  |  |  |  |  |  |  |  |  |  |  |  |  |  |  |  |
| 1925 | 4 | 12 | 4 | 31 | 12 | 12 | 4 | 4 | 4 | 6 | 6 |  | 6 | 6 | 6 | 8 | 10 |  |
| 1224 | 3 | 10 | 4 | 16 | 10 | 30 | 4 |  | 4 | 6 | 7 |  | 10 | 4 |  |  | 11 |  |
| 1283 | 2 | 20 |  |  |  |  |  |  |  |  |  |  |  |  |  |  |  |  |
| 1553 | 8 | 10 | 4 | 20 | 12 | 30 | 4 | 4 | 6 | 10 | 6 | 6 | 4 | 4 | 4 | 10 | 12 |  |
| 1713 | 3 | 20 | 3 | 10 | 8 | 30 | 12 | 4 | 6 | 7 | 6 | 10 | 6 | 5 | 5 | 10 | 6 | 10 |
| 1958 | 6 | 20 |  |  |  |  |  |  |  |  |  |  |  |  |  |  |  |  |
| 937 | 5 | 7 | 6 | 30 | 5 | 25 | 5 | 5 | 6 | 10 | 5 | 9 | 9 | 6 | 6 | 7 | 9 | 9 |
| 1928 | 9 | 21 |  |  |  |  |  |  |  |  |  |  |  |  |  |  |  |  |
| 1388 | 2 | 30 |  |  | 2 | 15 |  | 4 | 5 | 7 | 9 |  | 8 | 4 | 9 | 9 | 8 |  |
| 1637 | 4 | 30 | 4 | 15 | 4 |  |  |  | 10 | 7 |  |  |  | 7 |  |  | 10 |  |
| 1908 | 4 | 10 | 4 | 30 | 4 | 30 |  | 4 | 5 | 7 | 7 | 9 | 6 |  | 7 |  |  |  |
| 1459 | 4 | 25 |  |  |  |  |  |  |  |  |  |  |  |  |  |  |  |  |
| 1870 | 6 | 30 | 4 | 20 | 4 | 25 |  | 3 | 3 | 11 | 11 |  | 11 | 6 | 11 | 11 | 12 |  |
