# Supplementary material for: Potential association of vacuum cleaning frequency with an altered gut microbiota in pregnant women and their 2-year-old children
Source: Microbiome. 2015 Dec 21;3:65. doi: 10.1186/s40168-015-0125-2 (PMC4685603; doi:10.1186/s40168-015-0125-2)
Supplement: Additional file 2: Figure S1. — Out-of-bag evaluation of OTU importance by Random Forest for (A) mothers and (B) 2-year children. The models were based leave sizes of 10 for ensembles of 100 trees. The leave size was determined by the criterion of minimum mean squared error. (DOC 51 kb) [file 40168_2015_125_MOESM2_ESM.doc]

**Additional file 2. Supplementary Figures**


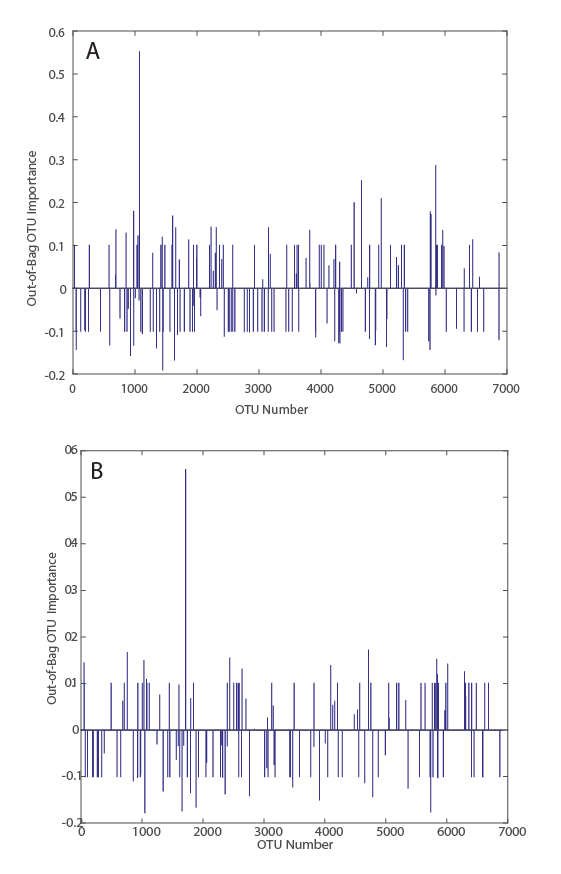


**Supplementary Figure 1. Out-of-bag evaluation of OTU importance by Random Forest for (A) mothers and (B) 2-year children.** The models were based leave sizes of 10 for ensembles of100 trees. The leave size was determined by the criterion of minimum mean squared error
